# Student’s Perspective and Teachers’ Metacognition: Applications of Eye-Tracking in Education and Scientific Research in Schools

**DOI:** 10.3389/fpsyg.2021.673615

**Published:** 2021-07-22

**Authors:** Raimundo da Silva Soares, Katerina Lukasova, Maria Teresa Carthery-Goulart, João Ricardo Sato

**Affiliations:** Center of Mathematics, Computing and Cognition, Federal University of ABC, São Bernardo do Campo, Brazil

**Keywords:** eye-tracking, teaching, learning, educational neuroscience, gaze, metacognition

## Abstract

This Perspective article discusses the possible contributions of eye-tracking (ET) to the field of Educational Neuroscience based on an application of this tool at schools. We sought to explore the teachers’ view of ET videos recorded while students solved mathematical problems. More than 90% of the teachers could predict with great accuracy whether the students had answered the questions correctly or not based solely on the information provided by the ET videos. Almost all participants tried to translate the students’ thoughts to understand the strategy used by the children. Our results highlight the relevance of qualitative analysis to identify the gaze strategies used by students. We propose that ET allows teachers to gain critical feedback about students’ behavior during problem-solving. Most previous studies tend to emphasize the benefits of ET applications to explore learners’ cognition. Our findings point that this system can also be useful to investigate teachers’ cognition by providing metacognitive experiences.

## Introduction

Scientific investigations in education can elucidate novel aspects of teaching-learning interactions and support evidence-based educational practices (Goswami, 2006; Szücs and Goswami, 2007; Dawkins and Epperson, 2014; Colvin, 2016). In this sense, technological tools used in scientific research, such as eye-tracking, could be useful in schools by providing relevant data on cognitive processes in different tasks, including learning performance (Hegarty et al., 1995; Van Gerven et al., 2004; van Gog and Scheiter, 2010; Jarodzka et al., 2013). Eye-tracking (ET) is a reliable option for research in schools as it provides a non-invasive and real-time measurement of performance by showing different strategies expressed by the participants during the task.

By analyzing the gaze behavior in pre-defined areas of interest (AOIs), it is possible to obtain relevant information on how they integrate knowledge and reason throughout the problem before selecting their answers in multiple-choice tests (Wang et al., 2014; Starr et al., 2018). ET also assesses cognitive processes and metacognitive strategies, which seems relevant for learning (Madsen et al., 2012; Paulus et al., 2013; van Gog and Jarodzka, 2013; Lin and Lin,2014a,b; Duchowski, 2017). Indeed, metacognition is a process of a higher level of reflection, observation, and regulation about one’s own learning (Flavell, 1979; Livingston, 2003). In addition, gaze patterns can inform about reading development and related impairments (Pavlidis, 1981; Eden et al., 1994; Rayner, 1998; Benfatto et al., 2016; Lukasova et al., 2016) and the development of numerical cognition and logico-mathematical skills (Schneider et al., 2008; Strohmaier et al., 2020).

ET also provides gaze replays available as videos that can be used as Eye Movement Modeling Examples (EMMEs). The video recordings of an expert’s eye gaze can be applied as an attentional pattern model to improve the learning of low-performing individuals (van Gog et al., 2009; Jarodzka et al., 2013; Mason et al., 2015). For example, Mason et al. (2016) demonstrated that young students with low reading comprehension skills improved their performance by observing the EMME of a high-performing student obtained while reading an illustrated text (Mason et al., 2016). Besides using ET as an intervention tool, it can also offer additional and refined ways to identify the strategies used in mathematical tasks and differentiate experts from those with difficulties. Students with math difficulties tend to show more diverse techniques than expert solvers. While using traditional methods to identify strategy pitfalls, such as “think aloud verbalization,” ET poses an advantage in doing so in a more natural and cognitively less demanding way, once transforming the math strategy into a verbal report may be overwhelming for low achieving students (Schindler and Lilienthal, 2018, 2019).

Studies on EMMEs made us wonder about how the gaze replay of students solving math problems could be informative for teachers. Despite this technology’s potential, it is not entirely known how teachers could use students’ eye-movement replay to improve their instructional methods. Although ET has the potential for education applications, it has not been widely used in the authentic school environment (van Gog and Scheiter, 2010; Mason et al., 2016). A profound discussion on how these technologies can be effectively applied in Education is needed and clarifies how teachers could benefit from this technology in teaching. A Perspective article is an excellent opportunity to motivate the debate and innovation around a specific topic or technology. In this sense, instead of presenting an evaluation through an experimental protocol, this study intends to discuss ET potential applications in educational contexts and interdisciplinary research on Educational Neuroscience. We hypothesize that teachers are not aware of how students’ gaze patterns can provide valuable information on problem-solving skills, and this tool may facilitate teaching and learning processes. Here, we provide an illustrative application focusing on these issues.

## Illustrative Application

This Perspective study was approved by Universidade Federal do ABC Ethics Committee. We obtained written informed consent from all adult participants and the parents/legal guardians of all non-adult participants that took part in the illustrative applications. Experiments were performed according to federal regulations and guidelines.

### Eye-Tracking Study

The participants (9 boys and 10 girls, 9–11 years old) from 5th grade in a primary school answered six mathematics multiple-choice questions (denoted by Q1 to Q6) from previous papers of the Brazilian basic education assessment system (SAEB, 2011). Previously to the questions, children were instructed on the protocol and underwent a gaze calibration process for the ET. The students could complete each math question (one per screen) at their own pace in the presence of one member of the research team that changed the slides after the child had verbally indicated the selected answer among available choices from four alternatives.

**FIGURE 1:**
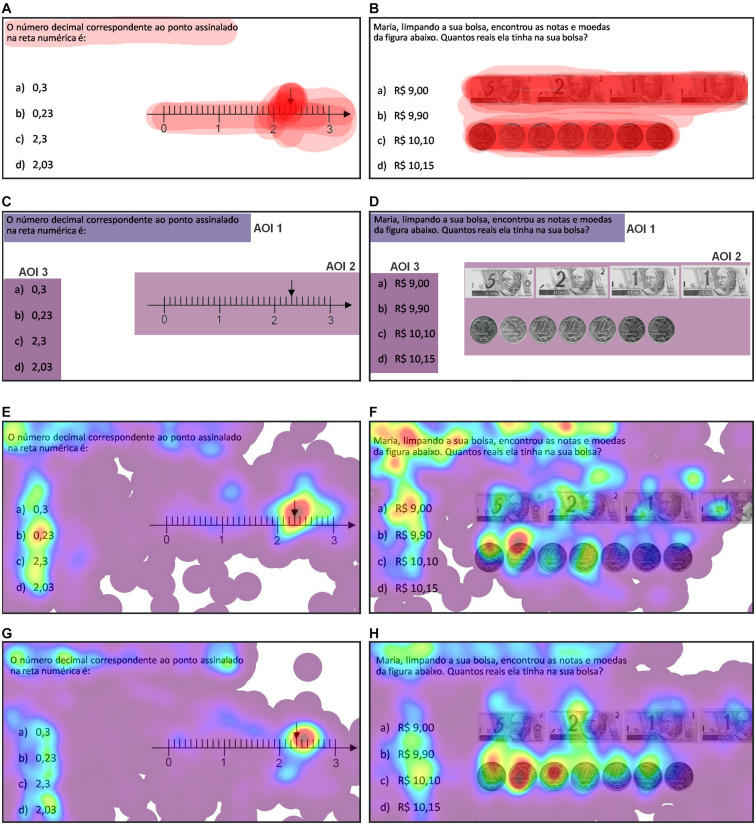
Eye-tracking graphical data. **(A)** Overlapping areas pointed out by teachers in Q1. **(B)** Overlapping areas pointed out by teachers in Q6. **(C)** Areas of interest in Q1. **(D)** Areas of interest in Q6. Problem statement (AOI 1), figure (AOI 2), multiple-choices (AOI 3). **(E)** Gaze heatmap of students that answered Q1 correctly. **(F)** Gaze heatmap of students that answered Q6 correctly. **(G)** Gaze heatmap of students that answered Q1 inaccurately. **(H)** Gaze heatmap of students that answered Q6 incorrectly. The red color in the heatmaps indicates more gazing time, and blue/purple indicates less gazing time from the students. The darker hues of red in the overlay result from the intersection of the areas pointed out by the teachers. Q1 problem statement: *The corresponding decimal number to the point marked on the number line is*. Q6 problem statement: *Maria, cleaning her purse, found the notes and coins in the figure below. How much money does she have.*

The eye-tracker (VT3-Mini 60 Hz, Mangold International GmbH., Arnstorf, Germany) was connected to the task presentation laptop. The Mangold Vision software was used on the laptop to display the stimuli and record the ET data, then analyzed by the Mangold Analyze software. We specified the area of interest (AOI) as the “problem statement” (AOI 1), “figure” (AOI 2), and all four “multiple-choice” (AOI 3), as shown in Figures 1C,D. The fixation count on each AOI was examined by averaging over the measures for all the students. We exported the heatmaps (Figures 1E,H), which show the spatial distribution of where the student was looked at the screen during the task (fixation times). The red spots in the heatmaps represent areas where the information was processed for a longer time by the participants, while the blue/purple color represents locations minimally explored.

After exporting ET videos of all students, for each question, we selected one video from a student with high performance and another from the child lowest performance (high- and low-performing students, respectively) as examples of the graphical data of gaze patterns that comprised ET videos (Supplementary Material). The illustrative applications of ET videos and the teachers’ views about students’ gaze patterns will be presented in the following subsections.

### Questionnaire With Teachers

This illustrative application’s main objective was to acquire insights about the qualitative information provided by the ET videos and heatmaps from the teachers’ perspectives. We interviewed 10 teachers from different schools where the data had been collected. After a brief explanation of the experiment, each participant was asked to point out the computer screen areas they thought students would look at more in each math problem. Then, we displayed the previously selected ET videos from the high and low-performing students solving the problems. The interviewed teachers had never met the children whose gazes were presented in the videos, and no information about their performances was provided. Teachers discussed two videos for each math problem presented (Q1–6) and answered nine questions (Supplementary Material). Responses were audio-recorded and transcribed for analysis.

Before the video presentation, teachers informed their opinion on “what is the region where students would look at most?” and if they considered the math problem “easy or difficult for a 5th grader in primary school.” After watching each ET video, teachers guessed if the student had chosen the correct alternative and what instruction they would give to that student to succeed in his choice in a new attempt to solve the problem. At the end of the questionnaire, teachers expressed if they were surprised and their opinion about ET videos relevance.

**FIGURE 2:**
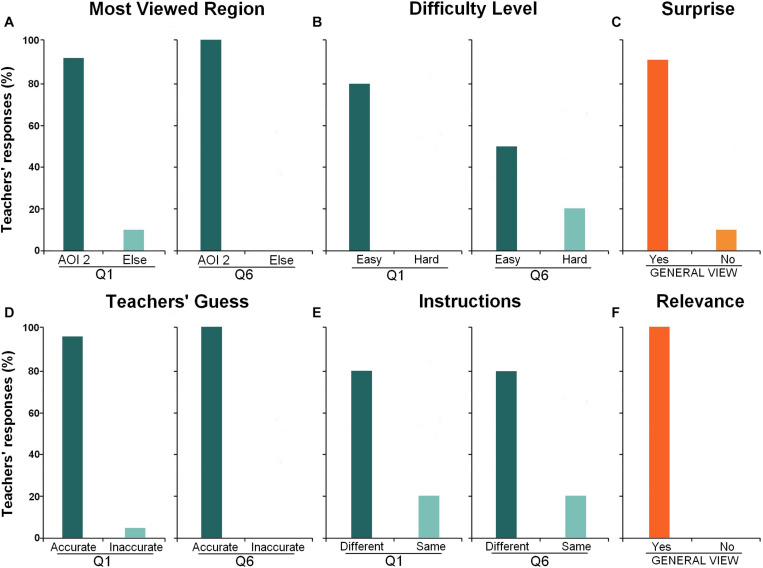
Teachers’ responses to the questionnaire. The bar graph represents the percentage of teacher responses in six categories: **(A)** most viewed region, **(B)** difficulty level; **(C)** any surprise; **(D)** teachers’ guess about student’s accurate or inaccurate answer; **(E)** the same or different instructions for both students, **(F)** eye-tracking relevance.

Based on the questionnaire, we grouped the teachers’ answers into the following six categories. (i) Most viewed region, (ii) Difficulty level, (iii) Any surprise, (iv) Teachers’ guesses about student’s correct, or incorrect responses, (v) The same or different instructions for both students, and (vi) Eye-tracking relevance. All responses were transcribed, translated (Portuguese-English), and analyzed using a series of categories and covering the teachers’ information. We followed systematic steps for data analysis based on the previous study (Campillo-Ferrer et al., 2020). By labeling six categories of teachers’ opinions, we analyzed the frequency of the most common answers for the content analysis calculated as percentages and presented in Figures 2A–F. We also marked the areas pointed out as “where students would look at more” in each mathematical problem and overlapped all figures to graphically demonstrate the teachers’ guesses (Figures 1A,B), which can be compared to students’ heatmaps (Figures 1E–H).

### Teachers’ View

The questionnaire results showed that 95% of the teachers were able to say whether the students answered the Q1 correctly or not based only on the information provided by the ET videos, and 100% of the teachers responded correctly about Q6 (Figure 2D). Also, almost all participants tried to translate the child’s gaze. After the first exercise, they focused less on the student’s last visualization and more on interpreting the ET pattern to understand the student’s thinking/way of reasoning.

*I was surprised because I was able to improve my perception by watching the child. Firstly, I thought that the question was easy, but then I saw it (the eye gaze) and thought to myself “hey, it’s not as easy as it seems”* (teacher 1).

In addition, 90% of the teachers expressed surprise about the students’ eye gaze behaviors (Figure 2C), and 80% considered Q1 easy, while 50% considered Q6 easy (Figure 2B). Also, 90% of teachers thought that students would look more at the figure in Q1, and 100% stated that students would fix their eyes more on the figure in Q6 (Figure 2A). Confronting the responses of how the teachers imagined the students’ behavior would be with the ET videos, it seems that some mistaken ideas surprised the teachers both about the students’ difficulties in solving the problems classified as easy/average and regarding the strategy expressed by the students.

*I imagined that they would read the questions less. I expected them to be more visual, but they were systematic. Even with doubts, they read the problem statement to get information and know the question* (teacher 5).

*It’s fascinating to see that He has got it right, but even more to know where his attention was* (teacher 2).

*Before watching this video, I didn’t think they would spend so much time reading the problem statement. I thought that they would spend more time on the image. It surprised me that the children start by reading the problem statement because it is something that we keep telling them to do. Now that we know that the child already focuses on the question statement, we can give new clues and try other interventions* (teacher 4).

*I think this student was more systematic in reading, but he must have been in doubt about the decimal number because he comes and goes several times* (teacher 5).

All teachers were able to identify difficulties in students’ solving skills, even when the children had chosen the right alternative. The high-performance student was “criticized” for not spending enough time reading the problem statement. The low-performance student’s gaze pattern was associated with a problem with reading and solving the maths operation. When asked about the instruction methods they would use with each student, most participants (80%) described different interventions for the high- and low-performance students considering the gaze behaviors (Figure 2E).

*One student did more or less what I imagined, but the other did not. They represent different styles of learning. I was surprised by one of the students that started by reading the alternatives to get the answer* (teacher 7).

Many teachers put themselves in the position of the students imagining that they were taking the test themselves to understand the strategies, even though not being asked to do so at any time during the interview.

*It’s because I’m very visual, so I always think the student would look at the image more* (teacher 1).

*I pretend that I am the student. It’s my assessment* (teacher 5).

All teachers reported that ET would be very useful in the classroom as a teaching aid tool because it allows a better understanding of students’ attention allocation patterns and of the possible different reasoning strategies used for problem-solving (Figure 2F).

*It is fascinating to see where the child is looking because we would know if what we propose is attractive. If we had access to this kind of information, we could give better classes* (teacher 9).

*You know students have mastered the content, but when they are put in a test situation, they don’t reach their full potential and make errors. It would be nice to bring this equipment to schools. It would help in designing the intervention* (teacher 10).

## Discussion and Perspectives

This Perspective article has demonstrated an illustrative application that explores ET’s use to record information about students’ gaze behavior while problem-solving. After applying an approach similar to what other research groups have already done focusing on students’ eye gaze patterns, we noticed that there could also be additional benefits for teachers in the development of some skills to analyze students’ cognitive processing to deliver increasingly better classes. First, we used ET to explore the gaze patterns on heatmaps and ET videos of high- and low-performing students solving mathematical problems. The heatmaps demonstrated that the overlapping areas pointed out by teachers in Figures 1A,B do not necessarily correspond to the regions viewed by the students. Our finding suggests that teachers did not know where students would look more when presented with a mathematics problem. Yet, we still needed to explore how the teachers’ view of ET videos was recorded while students resolved mathematical problems.

Curiously, most teachers were able to say with great accuracy whether the students answered the mathematical questions correctly or not based solely on the information provided by the eye-tracking videos. Also, almost all participants tried to translate the students’ thoughts to understand the children’s thinking. Although there are studies showing differences between people with high- or low-performance strategies during a reasoning task (Hegarty et al., 1995; Susac et al., 2014; Hu et al., 2017), none of the teachers had prior knowledge or guidance about any difference in eye-gazing patterns among students. The teachers’ experience in the classroom was possibly enough to interpret the strategy used by each student as a predictor of success or failure in problem-solving. Many teachers used the place where the student last looked in the video to hint at the student’s selected alternative. However, practically all teachers attempted to translate the students’ thoughts. After the first exercise, they focused less on the children’s last visualization and more on the interpretation of gaze patterns to understand the student’s strategy.

Teachers seemed to improve how they evaluated each video throughout this experiment, which favored a closer look at the aspects underlying the alternative’s students’ choice. It has been demonstrated that one-to-one assessment interviews can enhance teachers’ expertise because they can evidence how the children are thinking to solve an exercise (Bobis et al., 2005; Clarke et al., 2011). Here, the questionnaire used with ET seems to have a similar potential for schools’ applicability by providing insights into learners’ attainment. There is also the possibility of using gaze behavior in training programs to build an experience that may enhance expertise, particularly to novice teachers and those who have difficulties in this aspect.

All teachers could identify difficulties in students’ strategies, even when the children chose the correct answer. Teachers’ responses indicate that the high-performance student seemed to solve the mathematical problems very quickly with little attention to the problem statement. In contrast, the low-performance student had difficulties in both reading and specific mathematics contents. Such observations reveal that the grade does not necessarily represent performance. ET can facilitate interpreting the most prominent source/explanation for students’ difficulties and, without this equipment, some teachers would not have identified such problems. Many teachers also described different interventions for each student at the same mathematical problem. So ET videos provided an experience in which teachers could reflect on these different demands to help students overcome their difficulties. Many studies suggest eye movements analysis benefit a closer look at the aspects underlying the learning experiences and mathematical difficulties (Andra et al., 2009; Molina et al., 2018; Schindler and Lilienthal, 2018; Beach and McConnel, 2019; Sprenger and Benz, 2020; Strohmaier et al., 2020). Similarly, all teachers said that ET would be helpful in schools as a teaching aid tool because it allows for a better understanding of children’s attention patterns and the different reasoning strategies. It is a challenge to analyze the ET data in a class with many children. However, it is possible to apply this technology as an efficient screening method. The teacher could select a group of students as a sample to test hypotheses and better target interventions.

Some self-reported beliefs indicate a surprise by the teachers with children’s gaze. Indeed, we can see differences by comparing how the teachers imagined the students’ behavior with the ET videos. For example, students looked at all areas with information on the math problem (Figures 1E,H). In contrast, teachers believed that the children would explore more the visual information on the picture (Figures 1A,B), probably because that was where the teachers had looked at before watching the videos with the students’ gazes. Willingham (2017) argues that teachers could benefit by knowing the children’s cognition, emotion, and motivation, which he calls the “model of the learner” (Willingham, 2017). Similarly, our study suggest that teachers used themselves as a mental model to infer students’ behavior. However, by watching the gaze behavior, teachers re-evaluated their initial thoughts and may have enhanced their mental model of the learners. Therefore, ET could provide empirical observations to build the knowledge of what students are like and how they reason.

Several studies have shown that social-cognitive skills such as empathy, the theory of mind, and metacognition are associated with academic achievement (Chen et al., 1997; Malecki and Elliott, 2002; Kloo and Perner, 2008). Previous studies have highlighted the importance of metacognitive activities for success in the learning process (Wang et al., 1990; Veenman and Elshout, 1999; Susac et al., 2014; Muna and Bahit, 2020), such as mathematical solving-problems (Cohors-Fresenborg et al., 2010). Mok et al. (2006) demonstrated that teacher education students found the metacognitive approach supportive of their learning and self−assessment. In turn, ET may be a great tool to understand the teachers’ metacognition. There are studies exploring the possibility of applying EMMEs to improve their learning (van Gog et al., 2009; Jarodzka et al., 2013; Mason et al., 2015). Here, we emphasize the possibility of creating metacognition experiences for teachers to develop their instruction methods.

Although we only intended to present an illustrative application of ET videos as a perspective without an extensive experimental protocol, some limitations in the current article should be mentioned. A larger sample would be better to make the findings more conclusive, and a metacognitive assessment (Mok et al., 2006) could have been applied for a more objective analysis. The limitations suggest the opportunity for further studies, and the next step of our research is to investigate teachers’ metacognition development through students’ ET videos. Previous studies combined ET with neuroimaging techniques, such as fNIRS (Brockington et al., 2018) or fMRI (Lukasova et al., 2018), to investigate how students learn. However, there are questions to explore about the teaching process. Further research is necessary to elucidate the neural activity of the metacognition experience provided by ET. The more we understand teachers’ metacognition, the greater the chances of finding the best strategies to develop this teaching process.

We conclude that ET is a promising tool in the school context with applications that are also valid in the intrinsic scientific investigation of students’ and teachers’ cognitive and behavioral aspects. Children’s gaze data can help teachers characterize their students, which facilitates the interventions, and it can also create an experience that enables the development of teachers’ metacognition.

## Data Availability Statement

The original contributions presented in the study are included in the article/Supplementary Material, further inquiries can be directed to the corresponding author/s.

## Ethics Statement

The studies involving human participants were reviewed and approved by the UFABC. Written informed consent to participate in this study was provided by the participants’ legal guardian/next of kin.

## Author Contributions

RS and JS: designed the study, collected and analyzed the data, and wrote the manuscript. KL and MC-G: wrote and reviewed the manuscript. All authors have read and agreed to the published version of the manuscript.

## Conflict of Interest

The authors declare that the research was conducted in the absence of any commercial or financial relationships that could be construed as a potential conflict of interest.
