# Supplementary material for: Student’s Perspective and Teachers’ Metacognition: Applications of Eye-Tracking in Education and Scientific Research in Schools
Source: Front Psychol. 2021 Jul 22;12:673615. doi: 10.3389/fpsyg.2021.673615 (PMC8340918; doi:10.3389/fpsyg.2021.673615)
Supplement: Supplementary file 1 [file Data_Sheet_1.PDF]

Supplementary Material

1 Questionnaire

Questions repeated with each math problem presented

Most viewed region

In this question, what is the region where students would look at most?

Difficulty level

Do you consider this question easy or difficult for a 5th grader in primary school?

Teachers' guess about student's right or wrong answer

Based on this first video (low-performing student), do you believe that the student correctly answered the problem? Why?

Based on this second video (High-performing student), do you believe that the student correctly answered the problem? Why?

The same or different instructions for both students

Considering that he got it wrong (low-performing student), what instruction would you give him to answer correctly in a new attempt?

Considering that he got it wrong (high-performing student), what instruction would you give him to answer correctly in a new attempt?

Closing questions for the interview

Any surprise

Considering all the videos, was there anything that surprised you? Something you didn't expect?

Eye-tracking relevance

Do you consider videos like these useful for teachers? Why?

After this experience, is there anything you would reconsider from what you had answered before watching the videos?

Teachers' Answers to closing question for the interview

Question: After this experience, is there anything you would reconsider from what you had answered before watching the videos?

I thought the question was easy, huh? I expected students to be much quicker to answer because some questions weren't that difficult (teacher 3).

The last student had no idea how to count money (teacher 6).

I was surprised by, for example, when it comes to counting the coins and I couldn't touch them. I am not a very focused person so I got distracted. The student took as long as I did, but then he got it right (teacher 8).

It helps me think about students who don't learn. It would be very useful because it shows where I can improve my work with the student. It is a way for me to see where the student looks and then sit with him and help. I think it's amazing (teacher 1).

2 Eye-tracker default settings

We used 5 point calibration, which was informed to be sufficient by Mangold Vision Eye Tracking instructions. The same approach was applied in a previous study with children (Bolden et al., 2015). The mangold software grouped the raw data in fixation as follows: Cluster size (40 pixels) x Time window (1000 ms). In this default configuration, 40 pixels corresponds to one visual degree, which is customary in eye-tracking.
